# Transcranial magnetic stimulation combined with virtual reality improves quality of life in patients with Parkinson’s disease with depression

**DOI:** 10.3389/fneur.2025.1644962

**Published:** 2026-06-11

**Authors:** Ruiping Gu, Guizhen Zeng

**Affiliations:** Department of Neurology,Guangdong Neuroscience Institute, Guangdong Provincial People’s Hospital (Guangdong Academy of Medical Sciences), Southern Medical University, Guangzhou, Guangdong Province, China

**Keywords:** Parkinson’s disease with depression, transcranial magnetic stimulation, virtual reality technology, quality of life, depression and anxiety symptoms, motor function

## Abstract

**Purpose:**

To determine whether combining transcranial magnetic stimulation (TMS) with virtual reality (VR) provides superior improvement in quality of life, depression and anxiety symptoms, cognitive function, and motor performance in patients with Parkinson’s disease with depression (PD-D), compared with TMS alone.

**Methods:**

We prospectively analyzed 234 cases of Parkinson’s disease with depression (PD-D) admitted to our hospital from January 2022 to December 2024, of which 17 cases did not meet the inclusion criteria, 23 cases were excluded by the exclusion criteria, and 4 cases withdrew halfway, and finally 190 cases of PD-D were included. One hundred and ninety patients with PD-D as the subjects of this study. According to the patients’ treatment willingness, they were categorized into TMS group (TMS treatment, *N* = 106), TMS + VR group (TMS combined with VR treatment, *N* = 84). Clinical baseline data such as gender, age, body mass index (BMI), duration of PD, history of smoking, history of alcohol consumption, history of diabetes mellitus, history of hypertension, education level, place of residence, marital status, and H-Y staging were collected from all subjects.

**Results:**

Compared with the pre-treatment, the HAMA-14, HAMD-17, UPDRS-III scores and PDQ-39 total scores and their sub-scores decreased significantly in both groups after treatment, and their MoCA, MMSE, and ADL-BI scores increased significantly (all *p* < 0.05); and the magnitude of the changes in each of the scores was more pronounced in the TMS + VR group than in the TMS group (all *p* < 0.001). (all *p* < 0.001). However, there was no statistically significant difference in adverse effects between the two groups (*p* > 0.05).

**Conclusion:**

The combination of TMS and VR significantly improved the depression and anxiety symptoms, motor function and quality of life of PD-D patients, and the safety was good, which opened up a new path for personalized treatment of PD-D.

## Introduction

Parkinson’s disease (PD) is a neurodegenerative disorder characterized by degenerative lesions of nigrostriatal dopaminergic neurons as the core pathology, with a global prevalence of approximately 1–2%, and a significant increase in prevalence with age (1, 2). In addition to typical motor symptoms (e.g., resting tremor, myotonia, and bradykinesia), depression is a common non-motor symptom of PD, and may even precede the motor symptoms of Parkinson’s syndrome. It is estimated that about 40–50% of PD with depression (PD-D), which not only exacerbates motor dysfunction, but also significantly reduces the overall quality of life of the patients (3). The pathogenesis of PD -D has a complex pathogenesis involving synergistic dysregulation of dopaminergic, 5-hydroxytryptaminergic, and noradrenergic systems, as well as being closely related to functional abnormalities in the neural circuits of the cortico-basal ganglia-limbic system (4). Currently, clinical interventions for PD-D mainly include dopaminergic drugs, antidepressants and deep brain stimulation (5). However, medication is often limited by drug interactions and side effects (e.g., motor fluctuations, gastrointestinal reactions, susceptibility to drug resistance, etc.), and deep brain stimulation is difficult to popularize due to its high invasiveness and cost (6). Therefore, exploring non-invasive and multimodal synergistic treatment strategies has become an important research direction to improve the prognosis of PD-D patients.

Transcranial magnetic stimulation (TMS) is a non-invasive neuromodulation technique that uses rapidly changing magnetic fields to induce focal electric currents within the cerebral cortex. The induced currents transiently alter neuronal excitability and modulate functional connectivity across distributed brain networks, thereby influencing both local and large-scale neural dynamics (7). In recent years, the application of TMS in PD treatment has gradually expanded from motor symptoms to non-motor symptom management (8). Studies have shown that TMS significantly improves motor delays and gait deficits in PD patients, and the mechanism may be related to the enhancement of synaptic plasticity of cortical-basal ganglia loops (9). And studies have also shown that TMS also has a significant improvement effect on depression (10, 11). Meanwhile, TMS intervention can also alleviate depressive symptoms in PD patients by modulating 5-hydroxytryptaminergic transmission and Default Mode Network (DMN) activity (12).

Virtual reality (VR) technology, which provides patients with highly controllable simulation scenarios through multisensory immersive interactive environments, has demonstrated unique advantages in rehabilitation medicine, pain management, and psychiatric interventions (13). In the field of neurorehabilitation, VR can promote motor learning and balance function in Parkinson’s patients through task-oriented training; while in the treatment of psychiatric disorders, VR-supported exposure therapy (e.g., social anxiety disorder), cognitive-behavioral training (e.g., post-traumatic stress disorder), and mindfulness-meditation procedures are able to improve emotion regulation by remodeling abnormal neural representations (14). For example, “avatar” technology in VR environments can enhance patients’ self-identity through the mirror neuron system, while dynamic contextual simulation can help break the negative cognitive cycle in depressed patients (15). Notably, the real-time biofeedback function of VR can quantify patients’ behavioral and physiological responses (e.g., heart rate variability, eye movements), providing an objective basis for individualized treatment (16, 17). However, studies combining VR with neuromodulation techniques (e.g., TMS) are still in their infancy, and their potential synergistic mechanisms need to be explored.

Therefore, the present study aimed to determine whether combining TMS with VR yields superior clinical benefits over TMS alone in patients with PD-D. Specifically, we evaluated effects on quality of life, depressive and anxiety symptoms, cognitive performance, and motor function. This innovative interdisciplinary treatment paradigm not only provides a new treatment pathway for PD-D, but also opens up a new direction for other neuropsychiatric co-morbidities (e.g., Alzheimer’s disease with anxiety).

## Materials and methods

### Study population

We prospectively analyzed 234 cases of Parkinson’s disease with depression (PD-D) admitted to our hospital between January 2022 and December 2024. Of these, 17 patients did not meet the inclusion criteria, including 6 with Hoehn–Yahr stage >III, 4 with a prior PD-D diagnosis already receiving antidepressant therapy, 3 aged <18 years, 2 with significant communication disorders, and 2 with active suicidal ideation. Twenty-three patients met the inclusion criteria but were excluded based on the exclusion criteria: 7 with comorbid psychiatric disorders (e.g., schizophrenia, bipolar disorder), 5 with intracranial or internal metal implants contraindicating TMS, 3 with a history of epilepsy, 3 who had received electroconvulsive therapy or TMS within the past 3 months, 3 with severe cardiac, hepatic, or renal failure, and 2 who were pregnant or breastfeeding. An additional four patients who met all criteria withdrew during the intervention for personal reasons unrelated to adverse effects. Finally, 190 patients with PD-D were included in the study. After eligibility confirmation and baseline assessments, participants were offered both treatment options: TMS alone or TMS combined with VR. Assignment was non-randomized and based on patient preference following standardized counseling by the study neurologist, using a scripted description of potential benefits, risks, time commitment, and procedural differences. Preferences were elicited through structured discussion and documented in study records for traceability, with no coercion; undecided patients were encouraged to decide after Q&A to support shared decision-making in clinical practice. Those preferring VR were assigned to the TMS + VR group; others to TMS alone. To minimize expectancy and detection biases inherent in preference-based allocation, outcome assessors (independent neurologists not involved in counseling or treatment) were blinded to group assignment throughout evaluations. The study followed the ethical guidelines of the Declaration of Helsinki of the World Medical Assembly and the relevant norms and regulations of clinical research, and complied with the Enhancing the QUAlity and Transparency Of health Research (EQUATOR) network guidelines. The study was approved by the Academic Ethics Committee of our hospital, and all patients who participated in the study were fully informed of the purpose of the study and signed an informed consent form before sampling.

### Inclusion and exclusion criteria

Inclusion criteria: (1) patients who met the diagnostic criteria of PD-D and were diagnosed for the first time; (2) patients who met the indications for TMS treatment (18); (3) > 18 years of age; (4) those without suicidal behavior or suicidal ideation; (5) Hoehn-Yahr (H-Y) staging: stage I-III (19); and (6) those with no communication disorders.

Exclusion criteria: (1) patients with comorbid other psychiatric diseases; (2) patients with contraindications to TMS treatment, the presence of intracranial or internal metal implants, such as denture, intraocular metal implants, pacemakers, artificial valves, cochlear implants, etc.; (3) patients with organic brain disorders; (4) patients with a history of epilepsy or a family history of epilepsy; (5) patients with a history of drug dependence or abuse of antipsychotics; and (6) those who have received the past 3 months Electroconvulsive or transcranial magnetic therapy; (7) Pregnant or breastfeeding patients; (8) Patients with severe cardiac, hepatic, or renal failure.

### Sample and data collection

Clinical baseline data such as gender, age, Body Mass Index (BMI), duration of PD, history of smoking, history of alcohol consumption, history of diabetes mellitus, history of hypertension, education level, place of residence, marital status, and H-Y staging were collected from all subjects.

### Treatment program

#### TMS group

The TMS treatment plan was adopted, and a magnetic stimulator (MagPro R30, oniCA Elektronik A/S, Denmark) was used to stimulate the M1 area bilaterally, with a stimulation intensity of 90% of the resting motor threshold. The resting motor threshold was measured by first identifying the optimal stimulation site (“motor hotspot”) for the contralateral abductor hallucis muscle using standard procedures (51). The center of the “8”-shaped coil was initially placed over the estimated M1 area, and stimulation intensity was gradually increased to approximately 70% of maximum output for a single pulse while observing for visible contraction of the contralateral bunion. The hotspot was defined as the scalp position that consistently produced the strongest muscle contraction in at least five out of ten stimulations. Once identified, the site was marked on a tight-fitting elastic EEG cap with reference to anatomical landmarks (nasion–inion line and interaural line) to ensure reproducible coil placement across all sessions. The coil handle was oriented at approximately 45° to the sagittal plane to induce a posterior–anterior current flow, in accordance with established therapeutic TMS guidelines (20). At each session, the same trained TMS technician positioned the coil using the marked EEG cap, verifying alignment before stimulation to minimize inter-session variability (21). The stimulation frequency was 10 Hz, the stimulation time for a single sequence was 2 s, the interval time was 10 s, and the total number of pulses was 2000. Each treatment lasted 20 min (10 min on each side), and the device was automatically timed. Stimulation was performed 5 times per week for 4 consecutive weeks. High-frequency (10 Hz) was selected because it enhances cortical excitability and has shown efficacy for both motor and depressive symptoms in Parkinson’s disease, consistent with prior trials and international guidelines.

#### TMS + VR group

VR therapy was initiated on top of the TMS group, with each VR experience lasting 20 min per participant. The virtual reality system (C-Mill VR, Motek Medical B. V., The Netherlands) consisted of a Wii box, a Wii controller and a Wii Fit board. Three senior physiotherapists (movement specialists) selected programs for motor function, balance and activities of daily living (ADLs) based on a previous systematic study (22), including tennis, boxing, bowling, kicking, soccer, tilt slide, tilt city, trunk twisting exercises, and gait training (19). The training was done five times a week for four consecutive weeks of treatment.

### Concomitant treatments

All participants continued their standard Parkinson’s disease pharmacotherapy at stable doses for at least 4 weeks before enrollment. Patients on antidepressants were excluded at screening, per eligibility criteria. Other non-contraindicated medications and supportive therapies (e.g., physiotherapy) were permitted if stable and unchanged during the trial. Concomitant treatments were documented and monitored, and no systematic between-group differences were observed.

### H-Y stage

The H-Y stage of PD patients is 0–5, the higher the stage, the worse the symptoms. Stage 0: the patient has no symptoms; stage 1: the patient has unilateral body involvement; stage 2: the patient has symptoms on both sides, but balance is not affected; stage 3: the patient’s balance is affected, and the patient can stand and live on his/her own; stage 4: the patient’s voluntary activity is severely affected; stage 5: the patient cannot stand and walk without external help, and can only move around by a wheelchair (23).

### Hamilton Anxiety Rating Scale-14 (HAMA-14)

All subjects were assessed for anxiety using the HAMA-14 before and after treatment, respectively. The HAMA-14 is one of the most commonly used measures of anxiety by clinicians. It is a reliable and valid measure of the level of anxiety in depressed patients and has become the standard in the field. A score of <7: no anxiety present; 7–14: possible anxiety present; 15–21: anxiety present; 22–29 presence of significant anxiety; >29 severe anxiety. The higher the score, the more severe the anxiety disorder, totaling 56 points (24).

### Hamilton Depression Scale-17 (HAMD-17)

Depressive symptoms were assessed using the 17-item Hamilton Depression Rating Scale (HAMD-17) in its standard validated format. Assessments were performed at baseline and after completion of the 4-week intervention in a structured clinical interview by the study clinicians. Eight items were scored on a 0–4 scale and nine items were scored on a 0–2 scale, yielding a total score range of 0–52, with higher scores indicating greater depressive severity. The HAMD-17 has demonstrated good internal consistency (Cronbach’s *α* 0.70–0.79), high inter-rater reliability (ICC > 0.85), and concurrent validity in Parkinson’s disease populations. All assessments were performed by evaluators blinded to treatment allocation, following standardized administration and scoring procedures.

### Montreal Cognitive Assessment (MoCA) scale

MoCA is the most commonly used test for cognitive impairment in the clinical and research field, with a maximum score of 30, ≥26 indicating normal cognitive functioning, 21–25 indicating moderate cognitive impairment, and 0–20 indicating severe cognitive impairment, with the higher the score, the better the cognitive functioning (25).

### Mini mental state examination (MMSE)

MMSE was used to assess the cognitive status of all subjects before and after treatment, respectively. MMSE is also a commonly used clinical test for cognitive impairment, with a maximum score of 30, 27–30 indicating normal cognition, 23–26 indicating mild cognitive impairment, and the higher the score, the better the cognitive functioning (26).

### Unified Parkinson’s Disease Rating Scale part-III (UPDRS-III)

The UPDRS-III was used to assess the motor status of all subjects before and after treatment, respectively. The UPDRS-III score involves 14 entries, each on a scale of 0–4, with the highest score being recorded as 68, and the higher the score measured, the more severe the somato-motor symptoms (27).

### Activities of Daily Living-Barthel Index (ADL-BI)

The Activities of Daily Living-Barthel Index (ADL-BI) was administered to evaluate functional ability before and after treatment. Scores of 21–40 indicate marked dependence on assistance for daily activities (severe disability), while scores of ≤20 indicate complete dependence on others for daily living (total disability) (28).

### The 39-item Parkinson’s disease quality of life questionnaire (PDQ-39)

The PDQ-39 was used to assess the quality of life of all subjects before and after treatment, respectively. In clinical trials of PD, the PDQ-39 is the most widely used patient-reported scale, including Mobility (10 entries), Activities of daily living (6 entries), Emotional well being (6 entries), Stigma (4 entries), Social support (3 entries), Cognitions (4 entries), Communication (3 entries), and Bodily discomfort (3 entries), and other 8 dimensions (39 entries), with a score range of 0–4 points for each entry and a total score of 0–156 points, with higher scores indicating poorer quality of life for PD patients (29).

### Follow-up

The occurrence of adverse reactions (nausea, vomiting, dizziness, headache, scalp tingling, tinnitus, etc.) during the treatment period was recorded for each group.

### Statistical analysis

Data were statistically analyzed and graphed using SPSS statistical software (21.0, SPSS, Inc., Chicago, IL, USA), GraphPad Prism software (8.0.1, GraphPad Software Inc., San Diego, CA, USA). The Kolmogorov–Smirnov test was used to test for normal distribution; normally distributed measures were expressed as mean ± standard deviation; independent samples *t*-test was used to compare between groups; and paired *t*-test was used to compare measures before and after treatment. Non-normally distributed measures were expressed as median (very small value, very large value), and comparisons between groups were made using the Mann–Whitney U test, and before and after treatment using the Wilcoxon matched-pairs signed rank test. Count data between groups were expressed as the number of cases (percentage), and the chi-square test was used. The test level a = 0.05, P was two-sided test, and *p* < 0.05 was considered statistically significant difference.

## Results

### Characteristics of baseline data of the enrolled population

A prospective analysis of 190 cases of PD-D admitted to our hospital between January 2022 and December 2024 was performed, and patients were categorized into TMS group (TMS treatment, *N* = 106), and TMS + VR group (TMS combined with VR treatment, *N* = 84), according to their willingness to be treated. Clinical baseline data such as gender, age, BMI, duration of PD, history of smoking, history of alcohol consumption, history of diabetes mellitus, history of hypertension, education level, place of residence, marital status, and H-Y staging were collected from all subjects and analyzed, and the results showed (Table 1). Disease severity was assessed using the H–Y scale: 52 patients (27.4%) were stage I, 121 (63.7%) were stage II, and 17 (8.9%) were stage III. The distribution of severity did not differ significantly between groups (χ^2^ = 1.925, *p* = 0.382). In addition, all participants maintained stable Parkinson’s disease pharmacotherapy and supportive therapies for at least 4 weeks prior to enrollment, and no systematic differences in concomitant treatments were observed between groups. Comparative analysis showed no statistically significant differences across baseline variables between the two groups (all *p* > 0.05).

**Table 1:** General information of the enrolled population.

Project	TMS (*N* = 106)	TMS + VR (*N* = 84)	*z/t/x* ^2^	*p*
Sex (m/f)	68/38	57/27	0.286	0.593
Age (years)	60.81 ± 4.73	60.69 ± 4.29	0.182	0.856
BMI (kg/m^2^)	23.49 ± 3.69	23.34 ± 3.27	0.279	0.781
PD duration (years)	2 (1, 4)	2 (1, 4)	0.071	0.947
Smoking history (*N*, %)			1.154	0.283
Yes	41 (38.68%)	39 (46.43%)		
No	65 (61.32%)	45 (53.57%)		
Drinking history (*N*, %)			0.379	0.538
Yes	36 (33.96%)	25 (29.76%)		
No	70 (66.04%)	59 (70.24%)		
History of diabetes (*N*, %)			0.950	0.330
Yes	18 (16.98%)	19 (22.62%)		
No	88 (83.02%)	65 (77.38%)		
History of hypertension (*N*, %)			0.847	0.357
Yes	30 (28.30%)	29 (34.52%)		
No	76 (71.70%)	55 (65.48%)		
Educational level (*N*, %)			0.426	0.514
Secondary school, junior high school and below	68 (64.15%)	50 (59.52%)		
High school and above	38 (35.85%)	34 (40.48%)		
Residence (*N*, %)			0.574	0.449
Countryside	50 (47.17%)	35 (41.67%)		
Municipalities	56 (52.83%)	49 (58.33%)		
Marital status (*N*, %)			1.561	0.212
Divorced, unmarried, widowed	22 (20.75%)	24 (28.57%)		
Married	84 (79.25%)	60 (71.43%)		
H-Y staging (*N*, %)			1.925	0.382
Phase I	32 (30.19%)	20 (23.81%)		
Phase II	63 (59.43%)	58 (69.05%)		
Phase III	11 (10.38%)	6 (7.14%)		

TMS, Transcranial magnetic stimulation; VR, Virtual reality; BMI, Body mass index; PD, Parkinson’s disease; H-Y, Hoehn-Yahr; normal distribution test was performed using the Kolmogorov–Smirnov test for normal distribution, normal distribution of measurement data expressed as mean ± standard deviation, and comparison between groups using independent samples *t*-test; non-normally distributed measurement data expressed as median (very small, very large), and comparison between groups using the Mann–Whitney U test; between-group counting data expressed as the number of cases (percentage). Chi-square test was used.

### Depression and anxiety symptoms before and after treatment in each group

**Figure 1:**
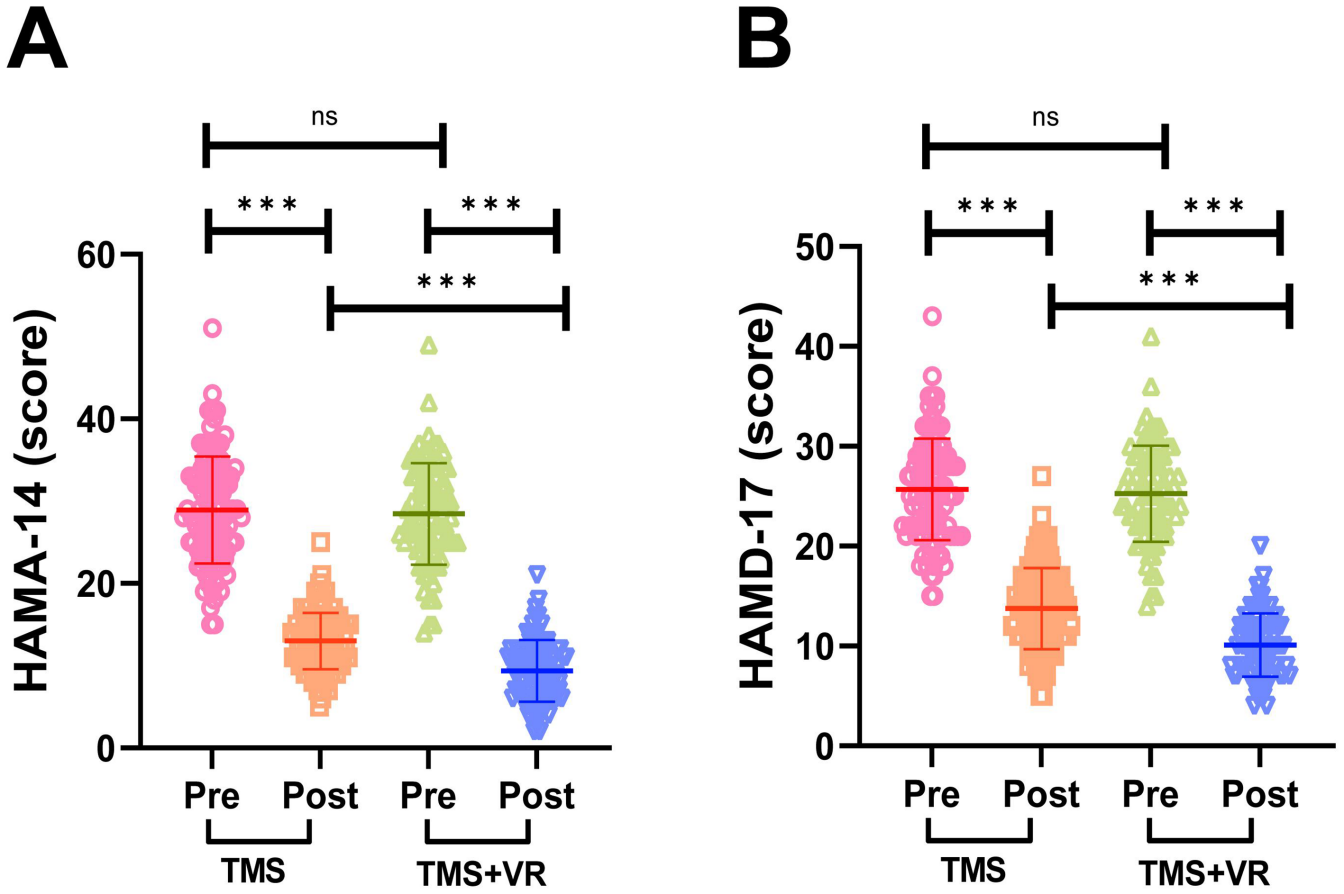
Effects of intervention on anxiety and depression scores. **(A)** HAMA-14 scores pre- vs. post-intervention; **(B)** HAMD-17 scores pre- vs. post-intervention.

We used HAMA-14 and HAMD-17 to assess the depression and anxiety symptoms of PD-D patients before and after treatment. The results showed (Figure 1): compared with the pre-treatment, the HAMA-14 and HAMD-17 scores decreased significantly in both groups after treatment; moreover, compared with the TMS group, the TMS + VR group HAMA-14 and HAMD-17 scores decreased significantly (both *p* < 0.001). It is suggested that TMS combined with VR can significantly improve the situation of depression and anxiety symptoms in PD-D patients.

### Cognitive and mental status before and after treatment in each group

**Figure 2:**
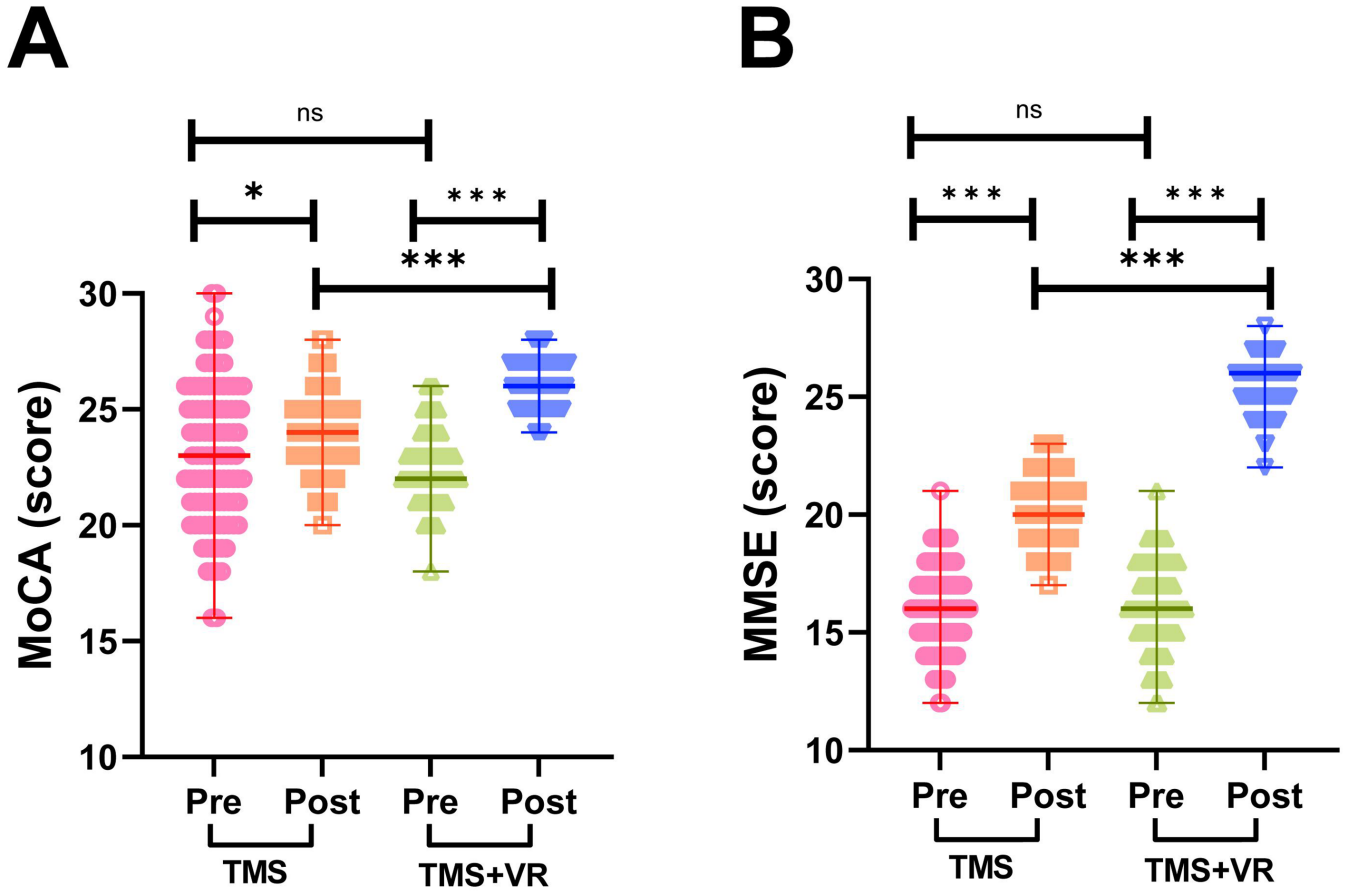
MoCA (Montreal Cognitive Assessment); MMSE (Mini-Mental State Examination); normal distribution tested using Kolmogorov-Smirnov test. Non-normally distributed measurements are expressed as median (minimum, maximum). Between-group comparisons used Mann-Whitney U test; **(A)** MoCA score comparison; **(B)** MMSE score comparison. *: *p* < 0.05; ***: *p* < 0.001; ns: *p* > 0.05.

We used MoCA and MMSE to assess the cognitive and mental status of PD-D patients before and after treatment, and the results showed that (Figure 2): compared with the pre-treatment, the MoCA and MMSE scores of the two groups were significantly higher after treatment; moreover, compared with the TMS group, the MoCA and MMSE scores of the TMS + VR group were significantly higher (both *p* < 0.05). It is suggested that TMS combined with VR can significantly improve the cognitive and mental status of PD-D patients.

### Motor function before and after treatment in each group

**Figure 3:**
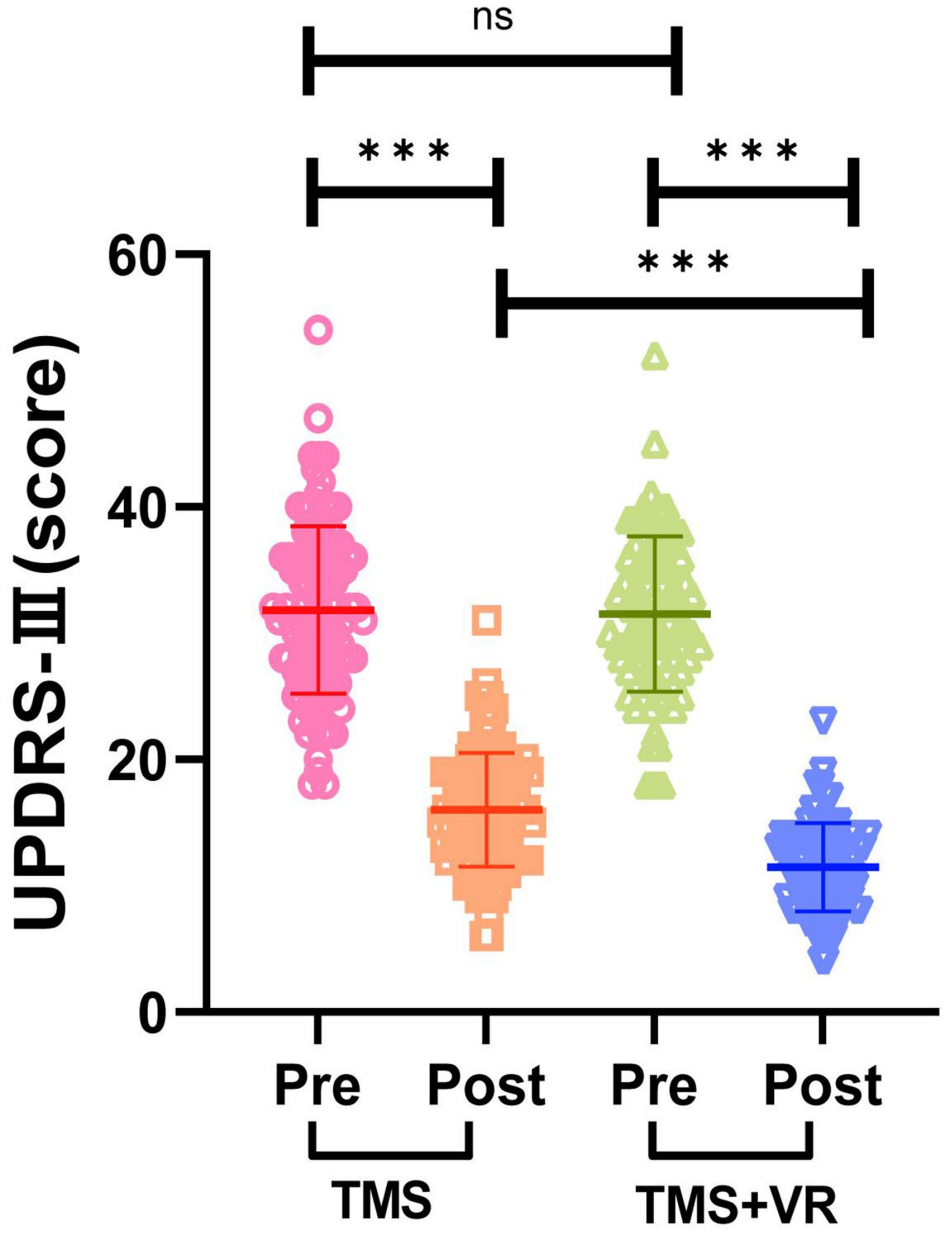
UPDRS-III scores before and after treatment in each group. Unified Parkinson’s Disease Rating Scale part-III (UPDRS-III). The Kolmogorov–Smirnov test was used to test for normal distribution; normally distributed measures were expressed as mean ± standard deviation, and independent samples *t*-test was used for between-group comparisons; ***: *p* < 0.001; ns: *p* > 0.05.

We used UPDRS-III to assess the motor function of PD-D patients before and after treatment, and the results showed (Figure 3): compared with the pre-treatment, the UPDRS-III scores of the two groups decreased significantly after treatment (both *p* < 0.001); moreover, compared with the TMS group, the UPDRS-III scores of the TMS + VR group decreased significantly (both *p* < 0.001). It is suggested that TMS combined with VR has significant efficacy in improving motor function in PD-D patients.

### Daily living ability before and after treatment in each group

**Figure 4:**
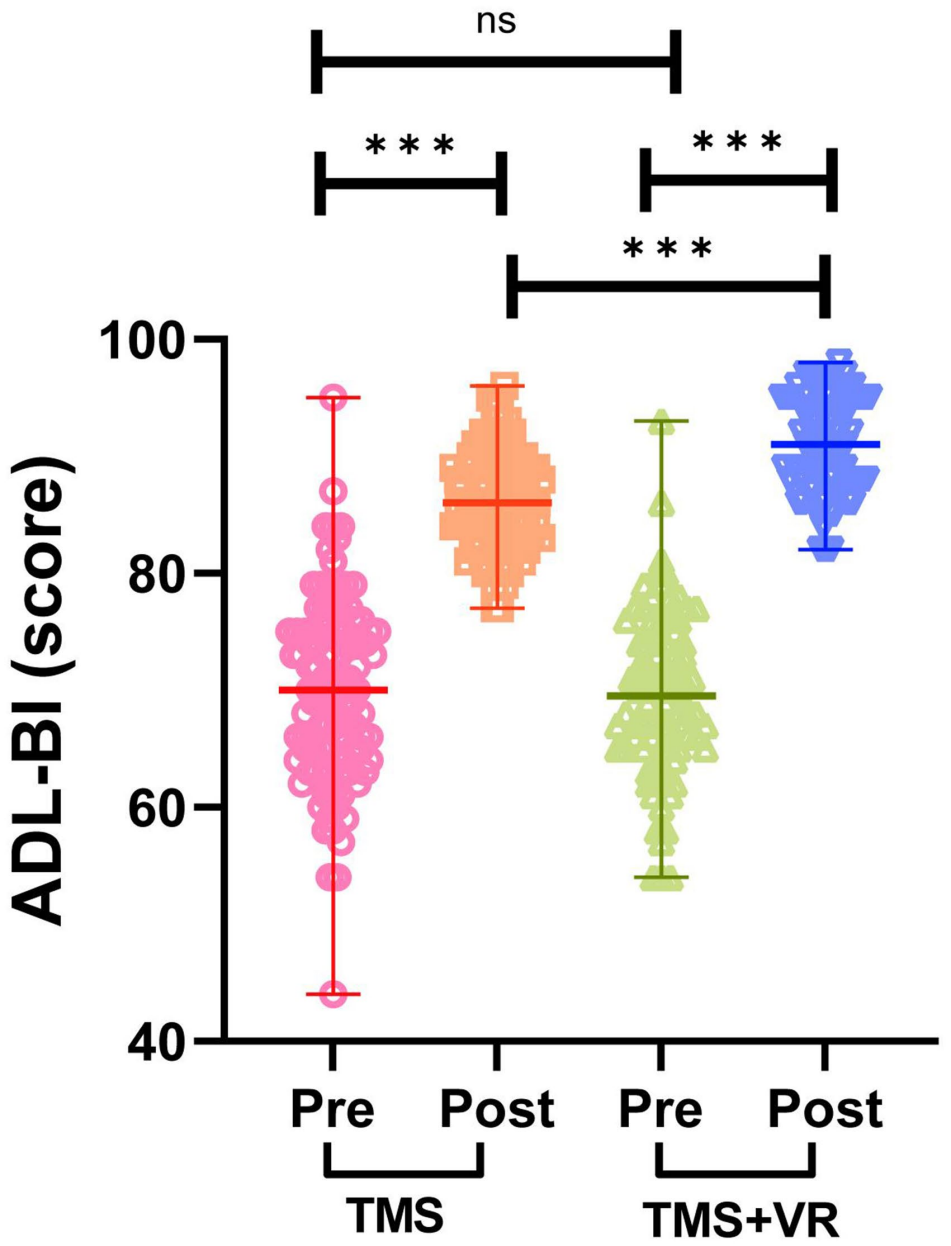
ADL-BI scores before and after treatment in each group. ADL-BI (Unified Parkinson’s Disease Rating Scale part-III). The Kolmogorov–Smirnov test was used to test for normal distribution, non-normally distributed measures were expressed as median (very small, very large), and the Mann–Whitney U test was used for between-group comparisons; ***: *p* < 0.001; ns: *p* > 0.05.

We used ADL-BI to assess the ability to perform daily living activities in PD-D before and after treatment, and the results showed (Figure 4): compared with the pre-treatment, the ADL-BI scores of the two groups were significantly higher after treatment; moreover, the ADL-BI scores of the TMS + VR group were significantly higher compared with those of the TMS group (both *p* < 0.001). It is suggested that TMS combined with VR can significantly improve the daily living ability of PD-D patients.

### Quality of life before and after treatment in each group

**Figure 5:**
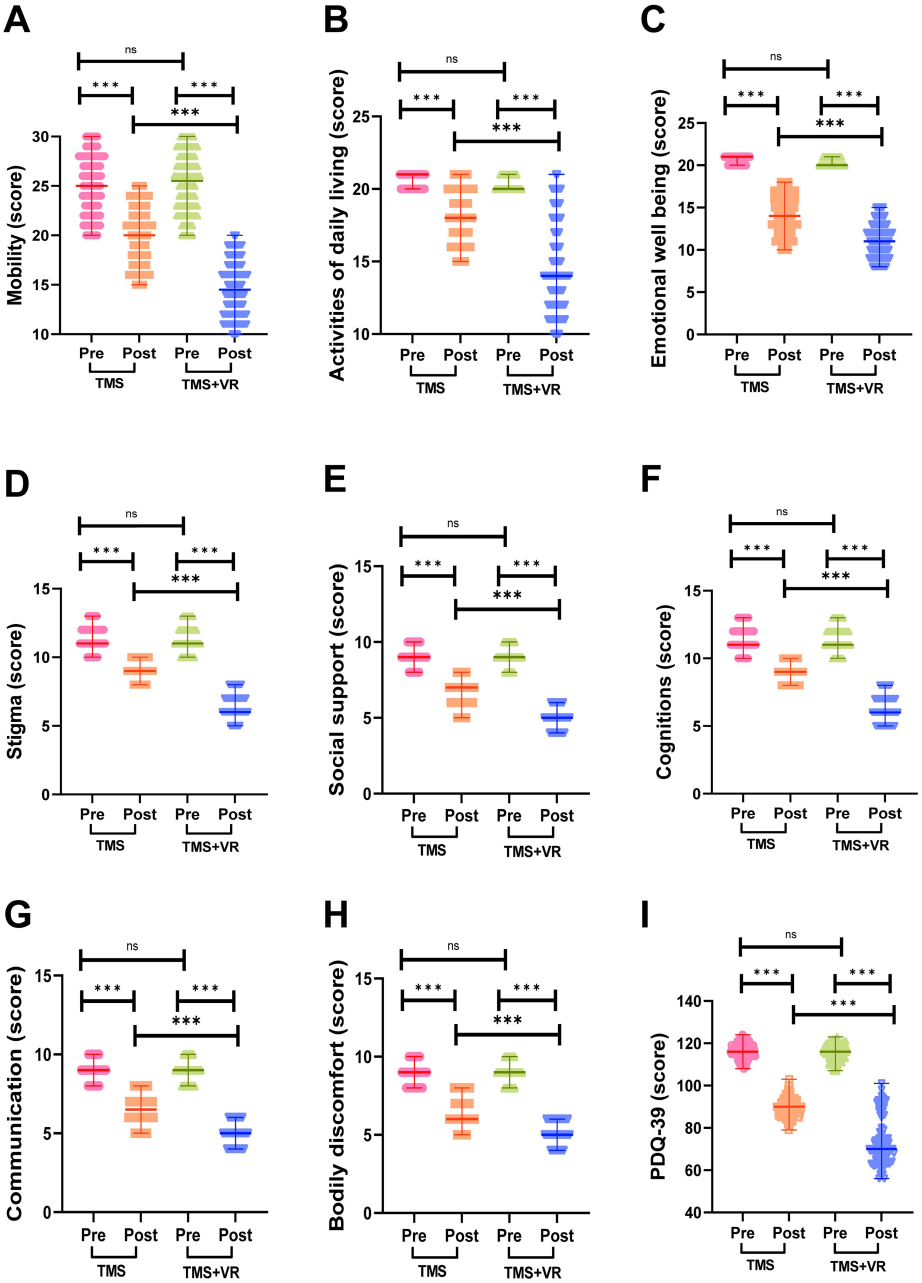
PDQ-39 scores before and after treatment in each group. **(A)** Mobility; **(B)** activities of daily living; **(C)** emotional well being; **(D)** stigma; **(E)** social support; **(F)** cognitions; **(G)** communication; **(H)** bodily discomfort; **(I)** PDQ-39 scores; normal distribution test using Kolmogorov–Smirnov test; non-normally distributed measures were expressed as median (very small, very large), and intergroup comparisons were made using the Mann–Whitney U test; ***: *p* < 0.001; ns: *p* > 0.05.

We used the PDQ-39 to assess the quality of life of PD-D patients before and after treatment, and the results showed (Figure 5): compared with the pre-treatment, the PDQ-39 scores of all dimensions in the two groups after treatment (Mobility, Activities of daily living, Emotional well being, Stigma, Social support, Cognitions, Communication, and Bodily discomfort) and their total scores were significantly decreased; moreover, compared with the TMS group, the PDQ-39 scores of all dimensions and their total scores of the TMS + VR group were significantly decreased (all *p* < 0.001). It is suggested that TMS combined with VR can significantly improve the quality of life of PD-D patients.

### Adverse reactions in each group

The occurrence of adverse reactions (nausea, vomiting, vertigo, headache, scalp tingling, tinnitus, etc.) during the treatment period was recorded in each group, and the results showed that (Table 2): there were 1 case of nausea, 1 case of vomiting, 2 cases of headache, and 2 cases of tinnitus patients in the TMS group; there were 3 cases of vertigo and 1 case of vomiting in the TMS + VR group, and there was no statistical significance for comparing the differences of the adverse reactions in the two groups (*p* > 0.05). It is suggested that further combined VR on the basis of TMS will not increase the risk of adverse reactions, but VR treatment may cause adverse symptoms such as vertigo.

**Table 2:** Adverse reactions in each group.

Private	Nauseating	Vomiting	Nephritis	Headaches	Tingling in the scalp	Tinnitus	Total adverse reaction rate
TMS (*N* = 106)	1 (0.94)	1 (0.94)	0 (0.00)	2 (1.89)	0 (0.00)	2 (1.89)	6 (6.60)
TMS + VR (*N* = 84)	0 (0.00)	1 (1.19)	3 (3.57)	0 (0.00)	0 (0.00)	0 (0.00)	4 (4.76)
*x^2^*							0.076
*p*							0.783

Counting information between groups was expressed as number of cases (percentage) using the Chi-square test (Chi-square test).

## Discussion

Currently, the clinical diagnosis and treatment of PD-D still face many challenges, firstly, the symptoms of PD-D overlap with the manifestations of PD itself, such as motor retardation and emotional apathy, which are easy to be misdiagnosed or underdiagnosed, and secondly, the biomarkers of depression (e.g., serum inflammatory factor, cerebrospinal fluid neurotransmitter levels) lack specificity, and currently rely on the subjective scale for diagnosis, which has certain limitations (30). In terms of treatment, although traditional antidepressant drugs can partially relieve symptoms, they are limited by dopaminergic system disorders and drug interactions in patients with PD, and their efficacy is unstable and prone to cause gastrointestinal reactions, upright hypotension, and other adverse effects (31, 32). Non-pharmacological therapies are also widely used in the treatment of PDD, among which cognitive-behavioral therapy has a certain effect, but the requirements for patients’ cognitive function and adherence are high, and the clinical application is limited (33). In contrast, TMS utilizes pulsed magnetic fields that act on the central nervous system of the brain to alter the membrane potential of cortical nerve cells, causing them to generate induced currents that affect metabolism and neuroelectric activity in the brain, and has been widely used in the treatment of depression (34). Our study adopted a high-frequency (10 Hz) TMS protocol, consistent with prior PD trials and guideline recommendations supporting excitatory stimulation of M1 or DLPFC to improve both depressive and motor symptoms (18, 35). Although a recent systematic review reported benefits from both low- and high-frequency stimulation, with some evidence favoring low frequency (36), we selected high frequency based on its established excitatory profile and consistency with clinical practice. VR provides multisensory stimulation through immersive environments, and has demonstrated potential for improving motor function and emotion regulation (37). Based on this, the present study focused on exploring the intervention effects of the combined application of TMS and VR in PD-D (50).

The results of this study showed that compared with pretreatment, HAMA-14, HAMD-17, UPDRS-III scores and PDQ-39 total scores with their sub-scores decreased significantly after TMS treatment, and MoCA, MMSE, and ADL-BI scores increased significantly. TMS stimulates specific brain regions through pulsed magnetic fields, modulates local neuronal activity and trans-synaptic connections, and enhances the dorsolateral medulla oblongata prefrontal cortex and the functional connectivity of the limbic system (e.g., amygdala, hippocampus), promoting the release of dopamine and 5-hydroxytryptamine, and improving depressive mood and motor retardation (38, 39). Studies have shown that dopaminergic neuronal degeneration in PD-D patients not only affects the substantia nigra-striatal pathway, but also leads to dysfunction of the prefrontal-limbic loop, and TMS alleviates the vicious cycle of affective and motor symptoms by restoring the excitatory balance of this loop (40). In this study, UPDRS-III scores decreased significantly in the TMS group, which may be related to the restoration of motor cortex excitability and functional remodeling of the basal ganglia-thalamo-cortical loop (41, 42). Meanwhile, over-activation of DMN (involving posterior cingulate gyrus and medial prefrontal lobe) in PD-D patients was associated with self-referential thinking and negative emotions, and TMS improved depressive symptoms by suppressing the abnormal activity of DMN and decreasing rumination thinking (43, 44).

In addition, the results of this study also showed that further combination of VR with TMS was more effective in improving depression and anxiety symptoms, cognitive and mental status, motor function, daily living ability, and quality of life in patients with PD-D. Currently, VR is often used as a therapeutic tool in the field of psychology or psychiatry (45). Some studies have shown that VR is effective in improving anxiety by eliciting realistic responses to fearful stimuli, while recent advances in VR have demonstrated the potential ability of VR to address cognitive and functional deficits in dementia, and have also shown that virtual reality environments have the potential to alter depression, cognition, and even social functioning (46). VR provides visual, auditory, and proprioceptive feedback through immersive environments to stimulate patients’ s motor intention and initiative (47). The real-time feedback mechanism of VR reinforces motor learning and promotes neuroplasticity, while gait training in virtual scenarios enhances basal ganglia-cerebellar-motor cortex synergy and improves motor performance in PD patients (48). Studies have shown that VR alleviates social withdrawal in PD-D patients by activating the mirror neuron system and enhancing empathy and social engagement (49). The combined application of TMS and VR may produce a synergistic effect to achieve multimodal neural remodeling, in which TMS modulates cortical excitability from the “top-down” and VR provides multisensory input from the “bottom-up” to promote the functional integration of the cortical–limbic-basal ganglia network. The results of the present study also suggest that the combined effect is effective. The results of this study also suggest that the combined efficacy of VR is better than that of single TMS treatment, which provides a new idea for the integrated treatment of PD-D.

In addition, further combination of VR on top of TMS does not increase the risk of adverse effects, suggesting that the combined treatment regimen has some safety. However, adverse symptoms such as vertigo may occur with VR treatment. Technical deficiencies such as motion sickness and dry eyes are the main reasons for the occurrence of adverse symptoms such as vertigo with VR, a deficiency that leaves its widespread use in psychiatry yet to be realized. However, it is worth mentioning that VR systems provide virtual environments with well-controlled sensory stimulation, and in the future VR systems may also become an innovative clinical tool for patients with specific psychiatric symptoms. The use of VR also opens up new and wide-ranging opportunities for the treatment of psychiatric disorders.

The combination of TMS and VR significantly improves depression and anxiety symptoms, motor function and quality of life in PD-D patients through multi-target neuromodulation and immersive behavioral activation with good safety, which opens up a new path for personalized treatment of PD-D. However, there are some limitations of the study, (1) despite the strict inclusion criteria, variation in baseline depression severity among PD patients may have influenced treatment outcomes. While motor disease severity (H–Y stage) was comparable between groups, stratified analysis of depression subgroups was not performed and should be considered in future research; (2) PD-D is a chronic progressive disease, and this study lacks the tracking of efficacy durability, and further clarification of the duration of maintenance of the intervention effect is needed; (3) the current conclusions are based on the behavioral scale, and the future needs to be combined with functional magnetic resonance imaging, electroencephalography or molecular markers to explore the neurophysiological mechanisms. (4) Preference-based allocation may have introduced expectancy bias, where the patients choosing TMS + VR could anticipate greater benefit. However, this was mitigated by baseline equivalence across groups (Table 1, all *p* > 0.05), structured neutral counseling to standardize expectations, and assessor blinding. (5) This study did not include a sham TMS + sham VR control group, which limits control over placebo and nonspecific effects. The design focused on evaluating the added benefit of VR to standard TMS in a clinical context, and bias was mitigated through blinded outcome assessment and equivalent therapist contact time. (6) Moreover, outcomes relied on validated but subjective rating scales without objective physiological markers (e.g., EEG, neuroimaging, or biomarkers), underscoring the need for future studies incorporating neurobiological or digital biomarkers. (7) Although all participants maintained stable pharmacological regimens, and no group differences were observed in baseline concomitant treatments, residual confounding cannot be fully excluded. (8) Finally, follow-up was limited to the immediate post-intervention period, preventing conclusions about the durability of treatment effects. Future trials should incorporate randomized allocation, sham-controlled conditions, objective biomarkers, and extended follow-up to strengthen causal inference and assess long-term efficacy.

## Data Availability

The original contributions presented in the study are included in the article/supplementary material, further inquiries can be directed to the corresponding author.
